# 50 years of the first ethics commission in Germany: the Ulm Ethics Commission in an international perspective

**DOI:** 10.3389/fpubh.2023.1197065

**Published:** 2023-06-15

**Authors:** Florian Steger, Oxana Kosenko

**Affiliations:** Institute of the History, Philosophy and Ethics of Medicine, Ulm University, Ulm, Germany

**Keywords:** ethics commission, ethics, public health, Declaration of Helsinki, Germany

## Abstract

**Background:**

The creation of the legal framework to recognize rights of patients and participants in clinical trials began in Germany in the 19th century. However, the ethical review of medical research in terms of the protection of rights and welfare of human subjects has only become a widespread practice since the establishment of ethics commissions. The first ethics commissions emerged at the universities under the influence of the German Research Foundation. The widespread establishment of ethics commissions began in the Federal Republic of Germany in 1979, after the adoption of the recommendation of the German Medical Association for the establishment of ethics commissions.

**Materials and methods:**

We analyzed unpublished archival documentation of the Ethics Commission of the University of Ulm and evaluated it based on a thorough review of research works on the history of international and German ethics commissions. For the examination of the sources, we implemented the historical-critical method.

**Results:**

The first ethics commission in Germany was set up at the University of Ulm in 1971/72. The reason for that was that the German Research Foundation required grant applications for medical research involving human subjects to be reviewed by an ethics commission. Initially the commission was created at the Center for Internal Medicine and Pediatrics, its authority grew over time until in 1995 it became the central Ethics Commission for the entire University of Ulm. Before the adoption of the Tokyo revision of the Declaration of Helsinki in 1975, the Ulm Ethics Commission developed its own guidelines for the conduct of scientific investigations on humans based on international ethical principles.

**Conclusion:**

The Ethics Commission of the University of Ulm must have been established between July 1971 and February 1972. The German Research Foundation played a decisive role in the establishment of the first ethics commissions in Germany. The Universities had to create ethics commissions in order to be able to obtain additional funds from the Foundation for their research. Thus, the Foundation initiated the institutionalization of the ethics commissions in the early 1970s. The functions and composition of the Ulm Ethics Commission were similar to other initial ethics commissions of the time.

## 1. Introduction

In Germany, the necessity to inform the patient and to consider the patient’s consent as a condition for medical action was recognized in the second half of the 19th century. The year 1890 can be considered a milestone in the history of patients’ rights, when the conduct of clinical experiments on human beings became the subject of intense public discussions. In 1891 in response to public outrage over Robert Koch’s (1843–1910) experiments with tuberculin on prisoners, the Prussian Ministry of the Interior issued a circular prohibiting the use of tuberculin against the patient’s will in prisons (1). In 1892, Albert Neisser’s (1855–1916) syphilis experiments on women, including minors, without their consent became a public scandal (2). In 1894, an Imperial Court decision finally followed, which recognized the importance of the patient’s will for the legitimization of medical interventions. One of the consequences of ethical discussions and public criticism in the political press and parliament about the abuse of human beings can be found later in the instruction issued by the Prussian Ministry of Education for the heads of medical institutions in 1900. The document regulated medical research involving human subjects and it formulated ethical rules for the treatment of participants in medical experiments (3). A further major developmental step for the rights of trial participants can be described as a result of the so-called “vaccination catastrophe” of Lübeck in 1930 (4). There, BCG vaccination experiments were conducted on newborns without the informed consent of their parents, resulting in the death of 77 children. The next year, the Reichsminister of the Interior issued *Guidelines for Novel Therapeutic Treatment and for the Conduct of Scientific Experiments on Humans* (5).

Although all above-mentioned guidelines strengthened the rights of patients and subjects, including their right for self-determination, during the Nazi period the guidelines were ignored (6–10). The history of patients’ rights only finds its continuation as a result of the Nuremberg Doctors’ Trial (1946/47). In the Nuremberg Code of 1947, for the first time in the history of medicine, systematic and comprehensive guidelines were laid down to ensure the permissibility of medical experiments (11). As a consequence of the Nuremberg Code, the Declaration of Helsinki was adopted by the World Medical Association in 1964 as a guideline for medical research. Ethics commissions became legally binding after the adoption of the Tokyo revision of the Declaration of Helsinki in 1975. It regulated principles of biomedical research involving human subjects. It must be noted here that in many countries, institutions that examine the ethical acceptability of research projects involving human subjects are called ‘committees.’ However, in Germany the word ‘commission’ is generally accepted. In contrast to commission, ‘ethics committees’ in Germany are competent bodies at the hospitals and clinics that deal with case-related clinical ethical counseling as well as with the counseling in fundamental ethical questions. Therefore, to avoid confusion, we use the word ‘commission.’

The widespread establishment of ethics commissions began in the Federal Republic of Germany in 1979, after the German Medical Association (Bundesärztekammer) had recommended that the State Chambers of Medicine (Landesärztekammer) set up commissions to advise and assess ethical and legal aspects of research on humans. The Medical Faculty Association endorsed the recommendation for the medical faculties of universities in June 1979. In May 1983, the Working Group of Medical Ethics Commissions in the Federal Republic of Germany including West Berlin was founded in Münster. In May 1985, the German Medical Congress approved an amendment to the Professional Code of Conduct, according to which a doctor had to contact an appropriate ethics commission before carrying out an experiment on humans (12). Since then the development of ethics commissions has gone along two tracks: on one side at the faculties of the universities and on the other side at the state medical associations.

In the German Democratic Republic, standards for the preparation and conduct of clinical trials of medicinal products existed since the 1960s. The Central Evaluation Committee for Medicinal Products was responsible for the evaluation of clinical trials, but the review of their ethical aspects was not part of its tasks. In the 1980s, a central Working Group of Medical Ethics Commissions was established and local ethics commissions or ethics officers were appointed (13).

After the German reunification, since 1994 the German Medicinal Products Act (Arzneimittelgesetz) has contained the regulatory requirement for ethics commissions to be set up before clinical trials are conducted on human subjects. As a result, the ethics commissions developed an authority character in matters relating to the Medicinal Products Act, which these commissions have retained to this day. Since 1994, the same rule has been applied to the Medical Devices Act (Medizinproduktegesetz). In 2004, the 12^th^ Amendment the German Medicinal Products Act came into force, which was mainly concerned with the transposition of EU directives into the national law, in particular with regard to the application of good clinical practice to the conduct of clinical trials with medicinal products for human use. Major changes have also taken place in the legal basis for studies with medical devices and *in vitro* diagnostics in 2021 due to new European regulations. These regulations strengthen and standardize conformity assessment procedures, clinical investigations, and clinical evaluations of medical devices.

Although the decision to establish commissions to advise and assess ethical and legal aspects of research on humans in West Germany was recommended in January 1979, the first ethics commission had already been established there in the 1970s. What was the reason? Was it intrinsically motivated or extrinsically? Was it an isolated decision or did it affect other universities as well? What was the composition of the commission and its functions? Was the experience of establishing the Ethics Commission in Ulm unique or similar to that of other countries? Did the international discourse on bioethics have an impact in this regard?

In order to answer these questions, we structured our paper as follows. First, we focus on the reasons for setting up the first ethics commission in Germany. Then, we address the institutional aspects and working fields of the Ethics Commission of the University of Ulm. In the discussion, we evaluate these results from the international perspective of ethics development of the time.

## 2. Materials and methods

To prepare the paper, we analyzed unpublished archival documents and research works. The group of archival documents includes internal records of the Ethics Commission of the University of Ulm. They contain information about its establishment, composition and areas of work. The commission’s correspondence with the German Research Foundation is especially important in this regard. We evaluated archival records based on a thorough analysis of research on the history of international and German ethics commissions. To examine the sources, we implemented the historical-critical method, which includes the stages of acquisition of primary sources and research works, critical evaluation of the information contained in primary sources, and presentation of data in historical context in terms of objectivity and significance (14).

## 3. Results

### 3.1. The Ethics Commission at the University of Ulm

#### 3.1.1. Establishment of the Ethics Commission

According to the documents of the Ethics Commission of the University of Ulm, the first ethics commission in Germany was most probably founded between July 14, 1971 and the first half of February 1972 at the Center for Internal Medicine and Pediatrics of the University of Ulm (15). That Center consisted of the Department of Internal Medicine and the Department of Pediatrics. The medical Directors of the first department were: Prof. H. Ditschuneit, Prof. H. Heimpel, Prof. E. F. Pfeiffer, Prof. Thure von Uexküll. The medical Directors of the second department were: Prof. E. Kleihauer, since 1975 also Prof. J. R. Poley and Prof. W. Teller. The Ethics Commission was established at that Center for several reasons. On the one hand, the Center conducted research involving human subjects, which required an ethical review. On the other hand, the medical staff at the Center had comprehensive competence in ethics, given their work with both adult patients and juvenile ones, who represent a vulnerable group. Unfortunately, the internal documentation of this commission from 1971 to 1972 has been preserved only fragmentarily. A list of Senate Commissions, Committees and Representatives of the University of Ulm dated July 14, 1971, does not indicate an Ethics Commission, so it must have been established after July 14, 1971. Even a thorough research could not find any reference to an earlier record of the Ethics Commission. However, the first reference about the formation of the commission is found in connection with the funding regulation of the German Research Foundation, the largest research funding organization in Germany. The Foundation circulated its letter of December 1, 1971, requesting funding applications by March 1, 1972, for approximately 50 special research areas (Sonderforschungsbereich, SFB) in Germany that had already been approved but not yet included in funding. Thereby the Foundation made it a condition, that the applications for clinical research involving human subjects must be approved by an ethics commission before any application for funding could be submitted. That condition of ethical review indicated that the German Research Foundation was one of the first institutions recognizing the need for ethics in research involving human subjects. The Foundation obtained recognition of the Declaration of Helsinki in research projects where human clinical trials were to be conducted. Therefore, it asked special research areas to establish ethics commissions and gain initial experience (16). Universities, in turn, realized that without ethics it would be impossible to obtain funding, and thus to conduct clinical trials on human subjects. For the young University of Ulm, which was founded in 1967, the inflow of funds was important for research and establishing itself among other universities.

In February 1972, the University of Ulm applied to the German Research Foundation for funding for two special research areas: the SFB 112 ‘Cell System Physiology’ and the SFB 87 ‘Endocrinology’. Within the SFB 87 only fundamental research, i.e. research on animals, was planned, so it did not have any clinical trials on human subjects at the time. Unlike the SFB 87, the SFB 112 involved clinical trials on human participants. In the SFB 122, several clinical research projects were planned with the aim of improving therapeutic measures in the treatment of leukemia. At the same time, the pathophysiology of diseases of the hematopoietic cell renewal systems had to be investigated. The study of hematopoietic cell systems was necessary directly involving human subjects because animal experimental models had a limited informative value and not always allowed direct conclusions to be drawn about the situation in humans. For example, there are no animal experimental models that have sufficient pathophysiological similarity to the diseases observed in humans (15). The German Research Foundation aimed at implementing the principles of Helsinki Declaration in biomedical research in Germany. The representatives of the Foundation were afraid that the level of sensitivity, which was particularly high among the researchers in the post-war years, could be gradually going down. Moreover, for structural reasons, the influence of older researchers on the doings of younger ones could decrease. Thus, ethics commissions could become an important mechanism for ethical review of research involving human subjects (17). That is why in 1971 the German Research Foundation conditioned the funding of research projects involving human participants to their prior review by ethics commissions. They had to make sure that there were no ethical problems and that the rights of clinical trial participants were sufficiently protected. Thus, the Foundation demanded the clarification of ethical issues before granting approval the SFB 112. The question was namely, how far the ethical guidelines of the Declaration of Helsinki were followed with regard to the inclusion of patients in hematology research projects (15). Since university commissions did not exist at that time and universities needed research funding, such commissions began to be established. The first one of them was founded in Ulm in 1971/72, the second one - in Göttingen in 1973. Since the SFB 112 was integrated into the Faculty of Clinical Medicine and assigned to the Center for Internal Medicine, Pediatrics and Dermatology within the faculty, it is not surprising that the Ethics Commission was established at that Center. The Center for Internal Medicine and Pediatrics could also have been chosen as the chair because of the special role of pediatrics, which deals with vulnerable patients. Moreover, representatives of internal medicine and pediatrics could provide a diversity in ethical review given their experience in treating adult and adolescent patients. It seems that initially the work of the Ethics Commission at the Center for Internal Medicine and Pediatrics was primarily focused on the projects of the SFB 112, because the ethical reviews of other university research projects involving humans were voluntary (17).

The grant applicant for the SFB 112 was Professor Theodor M. Fliedner, a hematologist and a pioneer of stem cell research. Fliedner’s Department of Clinical Physiology at the Center for Basic Clinical Research was the first functioning department of the new university. He was one of the founding professors of the University of Ulm in 1967, which was called back then the University of Medical and Natural Sciences. When it was founded, hematology was stipulated by the state government as a clinical focus for Ulm. Initially, a Research Group for Clinical and Experimental Leukemia Research was established, and later Fliedner inspired the setting up of the special research area 112 ‘Hematology’. The acquisition of the so-called third-party funds was always important to him in order to be able to pursue research projects and to continue financing researchers (18). The Foundation of the SFB 112 was supported by the Rector of the University of Ulm Helmut Baitsch (1921–2007). As a senator and a member of the board of trustees of the German Research Foundation, he was one of the initiators of the special research areas established by the Foundation in the late 1960s (19).

#### 3.1.2. Guidelines for the Conduct of Scientific Investigations on Humans

In 1972, Fliedner sent a statement on the principles and implementation of clinical research projects to Baitsch. Fliedner had written that statement with the director of the Clinic for Internal Medicine at the University of Ulm Prof. Hermann Heimpel (1930–2014) and professor for Internal Medicine at the University of Munich Herbert Begemann (1917–1994). At the same time, Fliedner confirmed that before October 4, 1972 an ethics commission had worked “on a trial basis” according to those principles (15). He mentioned, that on the basis of the relevant literature and within the framework of the principles established by the World Medical Association in 1955, 1961, and 1965, the medical staff of the SFB 112 developed *Guidelines for the Conduct of Scientific Investigations on Humans*. According to them, research projects had to be assessed in terms of their defensibility based on six following principles:

1. *Equality*: no experiment should be attempted, proposed, or undertaken to which the experimenter would not also subject his or her relatives, next friends, and himself/herself;
2. *Valid consent*: the voluntary consent of the person on whom an experiment is to be conducted must be obtained;
3. *Prohibition of clinical trials involving illicit human subjects*: there are persons for whom the conduct of research studies cannot be ethically justified (e.g., mentally ill persons), since they do not provide a clear insight into themselves and their environment;
4. *Previous animal experiments*: in case of studies on human subjects, the safety or risk of experiment must have been adequately clarified in the animal experiment. In addition, it must be proven that scientific research relevant to humans can only be achieved through investigations on humans themselves;
5. *Competence and skill of the investigator*: basic clinical research may only be performed by appropriately qualified physicians;
6. *Accurate recording*: the investigator must keep accurate records of the clinical trial he/she conducts in order to keep the experiment completely transparent (15).

The hematological research projects of the SFB 112 respected those principles and took them fully into account during the planning of experiments. In December 1972, the German Research Foundation came to a positive decision on the funding for the SFB 112, but with one ethically relevant restriction. In the opinion of the Foundation, the issues discussed in the meeting of reviewers with regard to the repeated taking of tissue samples, the use of radioactive isotopes, and bacterial decontamination had not yet been satisfactorily clarified in all respects, despite the detailed statement written by Fliedner and his colleagues. The Foundation had therefore appointed a commission to discuss those issues. Until its final statement, which was expected in the spring of 1973, the clinical trials of ethical concerns (tissue samples, the use of radioactive isotopes, and bacterial decontamination) were not allowed to be carried out under the SFB 112. That meant a considerable restriction on research. Once again, Fliedner had to submit a detailed explanation. For that purpose, a working group ‘Ethical Issues in Clinical Research’ was formed. The aim of the working group was to develop guidelines for conducting scientific research on humans for submission to the Foundation, which could be applied not only to the SFB 112, but also to other special research areas in the future. In the fall of 1973, the Foundation finally dealt with the ethical issues and, on the basis of the explanations submitted by the SFB 112, lifted the restrictions. At the same time, the *Guidelines for the Conduct of Scientific Investigations on Humans* submitted by the SFB 112 were criticized, explicitly pointing out the inadmissibility of experimental interventions in children that were not medically indicated (20).

In the following years, those principles were applied to other research projects of the University of Ulm, in which human trials were conducted. Thus, the German Research Foundation initiated the institutionalization of the ethics commissions in the early 1970s, as the funding of new special research areas required to establish an ethics commission for research involving human subjects. That was long before the recommendation of the German Medical Association for the establishment of ethics commissions in Germany in 1979.

#### 3.1.3. Empowerment of the Ethics Commission

In 1976, the Dean of the Faculty of Theoretical Medicine Prof. Otto Haferkamp (1926–2016) approached the Rector of the University of Ulm Prof. Ernst Fr. Pfeiffer (1922–1997) with the proposal to establish a Commission for the Protection of Human Subjects in Scientific Studies, which was supposed to be responsible for the whole University (17). Thus, in 1977 the Senate Commission ‘Research on Humans’ was established with the responsibility for the entire University of Ulm. According to Fliedner, the previous Ethics Commission at the Center for Internal Medicine and Pediatrics no longer existed by 1978 (17). The director of the Department of Clinical Physiology Prof. Theodor M. Fliedner (1929–2015) was appointed the chairman and the director of the Pathology Institute Prof. Haferkamp became a vice chairman of the new Commission. It also included two clinicians - the director of the Clinic for Trauma Surgery Prof. Caius Burri (1930–2002) and the director of the Clinic for Internal Medicine Prof. Heimpel, − and the pharmacologist Prof. Hermann Bader (geb. 1927) (17).

The same year, 1977, the German Research Foundation discussed the recommendation of the Declaration of Helsinki, revised by the World Medical Association in Tokyo in 1975, to establish local ethics commissions. Therefore, the establishment of a university-wide Ethics Commission at the University of Ulm coincided with the proposal of the German Research Foundation. However, it soon turned out that the chairman of the Ethics Commission Fliedner understood its task as “to discuss the responsibilities, procedures and composition of a commission,” but not to discuss specific research applications. Thus, he offered to build an ‘*Ad Hoc* Committee Review of Clinical Research Projects.’ In 1978, such a committee, later known as the ‘Advisory Commission of the Small Senate’ was indeed established under the chairmanship of the Professor of pediatrics Kleihauer. As a result, at the beginning two ethics commissions with different tasks co-existed simultaneously. In addition to Kleihauer, the other members of the ‘Advisory Commission of the Small Senate’ were: clinicians Peter Merkle (surgery), Prof. Hans-Eduard Franz (nephrology) and Prof. Wolfgang Dick (anesthesiology); pharmacologist Prof. Bader; basic scientist Prof. Hans-Dieter Flad (microbiology). As non-medical members there were the hospital priest Wolfgang Lipp and the lawyer Ule Wulf (17). The subsequent experience of the Ethics Commission of the University of Ulm shows that it continued to consist of eight members, composed of four clinicians, a basic scientist, a pharmacologist, a lawyer, and a pastor. An innovation in the mid-1980s was involving a medical student in the work of the commission, who had no right to vote (16).

The commission reviewed numerous applications for which, as a rule, there were no ethical objections to the conduct of investigations. Frequently, questions of liability insurance, risk, especially in view of side effects, informed consent were raised. In 1988, for the first time, in view of the risk to the research subjects, an application to test recombinant human interferon on healthy young adults was rejected. After the detailed discussion, the Ethics Commission came to a conclusion that the gain in knowledge and the risks for the healthy research subjects were not in a justifiable relationship to each other (21). From January 1, 1987 to October 4, 1988, 70 applications were submitted to the Ethics Commission. Of those, three were rejected due to unreasonableness for the patient or for the volunteer, and in case of one application, jurisdiction was denied (21). Thus, in the late 1980s, about 35 applications per year were processed and about one to two applications per year were rejected. The Commission also expressed constructive criticism to some applicants so that their applications could be more clearly and unambiguously formulated and the clinical trial process could be accelerated.

Since 1979, ethics commissions have been anchored in the German medical professional law: in the Medical Professional Code of Conduct, a consultation of physicians by an ethics commission of the medical association or a medical faculty was initially optional and from 1988 it became mandatory. The pharmaceutical companies had to submit phase III drug studies to the university ethics commissions or those of the state medical associations - regardless of whether a member of the respective university was involved. The mandatory approval of clinical projects by the ethics commission led to an increase in applications to the Ethics Commission of the University of Ulm in 1989. While in 1987 the commission considered 33 applications and in 1988 41 respectively, in the first quarter of 1989 alone 17 applications were received (21). Thus, an optimization of the procedural flow in the work of the Ethics Commission was undertaken. A head office was established, and the Commission was allowed to charge a fee for its expertise (22). In 1989, the Senate Commission ‘Research on Humans’ was abolished and a central Ethics Commission of the Faculties of Medicine of Theoretical and Clinical Medicine was re-established instead. This means that all university members, including students working on a doctoral project, were obliged to consult the Ethics Commission in case of research involving human subjects, including epidemiological research (23).

Since 1994, the German Medicine Act has been the first statutory regulation to require the vote of ethics commissions prior to the conduct of a clinical trial of medicinal products on humans. As a result, the ethics commissions developed the character of authorities in matters relating to the drug studies. Since 1995, the Ethics Commission has been a central commission of the University of Ulm for all members of the university.

Since then, there have been few changes in the work of the commission, particularly with regard to its composition. Starting from 1991, a student could no longer be a member of the commission, and since 2016, the clergy could no longer participate in its work. The commission currently consists of 21 members who represent various branches of medicine, such as forensic psychiatry, internal medicine, pharmacology, psychology, human genetics, pediatrics, transfusion medicine, surgery, epidemiology, biometrics as well as lay persons and representatives from the medical ethics, nursing service and lawyers.

## 4. Discussion

As researchers suggest, the development of bioethics in the USA since the early 1960s as well as the establishment of ‘institutional review boards’ served as a model for the ethics commissions in Germany (24–27). However, this refers more to the period of the 1980s, whereas the 1970s are considered as experimental years in the creation of the very first German ethics commissions (28). Taking the Ulm Ethics Commission as an example of the first ethics commissions in West Germany, we aim at discussing the following aspects: (1) the reasons for the establishment of the first ethics commissions in Germany and other countries; (2) composition and competences of the first ethics commissions. These aspects will allow us to make a conclusion, if the reason for the establishment of the first ethics commission in Germany and its working principles were in line with the international development of ethics at the time, and if the experience of the US ethics commission influenced the German development.

### 4.1. Reasons for the establishment of the first ethics commissions

First, we want to discuss and compare reasons for the establishment of the first ethics commissions in other countries. As far as we know from the research literature, the first ethics commissions called ‘radioisotope committees’ and ‘subcommittees on human use’ were established in the United States in the late 1940s (17). The establishment of ethics commissions in Sweden in the 1960s followed at all medical faculties of universities. The widespread establishment of ‘ethics committees’ in the United Kingdom is characteristic of the second half of the 1960s and early 1970s. The first ethics commissions in Germany emerged in the early 1970s. In the late 1970s and mid-1980s, scientific journals published information on the establishment of ethics commissions in Sweden, Austria, Belgium, Denmark, Norway, Israel, Scotland, Canada, Australia, New Zealand, Japan and Malaysia (17).

Although in Germany it was known, that the first ethics committees were known to had been established in the USA, Sweden and the UK, the medical faculties of the German universities were hesitant to set up ethics commissions in the 1960s. The reason for that was probably that the laws determined actions of researchers, so they were responsible for conducting research involving human subjects, regardless of the control of the ethics commission. Moreover, as in any other field of science, medical researchers relied on the principle of self-control in conducting clinical trials. The requirement of the German Research Foundation for ethical review of projects submitted for funding to ensure the protection of human subjects’ rights correlates with the same initiative of the US Public Health Service. In 1966, the US Surgeon General William H. Stewart issued a directive related to investigations involving human beings. According to that directive, the funding for clinical research involving human subjects from the US Public Health Service could only be possible, if the applicants provide a prior ethical review of their projects. Such ethical aspects as the rights and welfare of the trial participants, their informed consent, risks and potential medical benefits of the clinical trials were specifically implied. As a result, the directive led to the creation of special research ethics committees at medical institutions that were recipients of the grants. The establishment of such committees in hospitals and medical schools took place not only in the United States, but also in the United Kingdom and Sweden, which also received funding from the US Public Health Service (29).

As the representatives of the German Research Foundation pointed out, they feared a decrease in the level of sensitivity of researchers in ethics issues in the post-war years. Thus, the implementation of the principles of Helsinki Declaration in biomedical research in the form of ethics commissions seemed to them an important mechanism for ethical review of research involving human subjects.

The Foundation’s request for the approval of the ethics commission before it provides the funding raises the question if the Foundation could control ethical conduct indirectly. Using the example of the Ulm Ethics Commission, we can see that the Foundation asked several times for a detailed clarification of the ethical issues before granting approval to the SFB 112. However, the provision for clarification was not due to a lack of elaboration on ethical issues, but due to the need to clarify the specificity and necessity of clinical trials involving human participants. We have no evidence that the Foundation interfered in the conduct of work of the SFB, including ethical issues. We also have no evidence that the Foundation did not approve any of the SFB projects for ethical reasons. We see the introduction of ethics commissions into the work of medical research institutions as a progressive step by the Foundation in establishing a mechanism for controlling good clinical practice.

Although the Foundation representatives were aware of the directive of the US Surgeon General in 1966, it is difficult to say with certainty that it had a direct influence on the similar Foundation requirement. It is likely that for Germany, the experience of Nazi medical crimes, including experimentation without the consent of human subjects, played a much more important role in the context. However, we can obviously say that the condition of ethical review of projects submitted for the German Research Foundation grants initiated the formation of the first ethics commissions in West Germany. As we could trace from the archival documents, the Ethics Commission of the University of Ulm was most probably founded no earlier than July 14, 1971 and no later than the first half of February 1972. According to the professor of pediatrics Enno Kleihauer (1927–2017), who was the chairman of the Senate Commission ‘Research on Humans’ at the University of Ulm, the Ethics Commission in Ulm was founded in 1971 (24).

### 4.2. Composition and competences of the first ethics commissions

The Ulm Ethics Commission has undergone several reorganizations and has gradually expanded its expertise or ethical review from research projects funded by the German Research Foundation to all university projects involving human beings. In the early years of its operation, the Commission was guided in its work by international ethical recommendations, particularly the principles established by the World Medical Association. The Commission even developed its own *Guidelines for the Conduct of Scientific Investigations on Humans* in 1972. This demonstrates that the Commission began its original way of working, without drawing on the experience of the US Institutional Review Boards, about whose work principles little was known at the time.

If we look at the composition of the first ethics commissions in Germany, the United States and Sweden, we can state that it was a sort of experiment in the beginning. In the United States or Sweden, the members of the commission could be not only physicians, but also theologians, biologists, psychologists, nurses and students. In the United States in the late 1970s, commissions consisted for the most part of clinicians and some representatives of the experimental sciences. The participation of an experienced lawyer was also valued (26, 30, 31). In the late 1970s, the practice of involving medical students in the work of ethics commissions was quite common. In 1978, the US National Commission for the Protection of Human Subjects of Biomedical and Behavioral Research issued recommendations that investigators and persons independent of their research area should conduct ethical reviews. The Commission justified its position by arguing that researchers are potentially in conflict by virtue of their pursuit of knowledge and concern for the welfare of trial participants. However, since involving lay people was particularly problematic when assessing risks and benefits, ethics commissions began to engage medical students in their work. Student members were considered as ‘informed outsiders’ who could bridge the gap between lay people and professionals. On the one hand, students had knowledge of basic scientific and medical concepts, and, on the other hand, they had more distance and less bias than investigators in the evaluations of risks to human subjects and benefits to the society (31). In Germany, the experiments lasted much longer than in the USA. In case of the Ethics Commission of the University of Ulm, the student was withdrawn from the commission in 1991 and the pastor in 2016. Women did not participate in the work of the commission back then.

There were different views on the competencies of the ethics commissions in three countries. While in the UK the review activities were explicitly limited to ethical issues, the US-American and Swedish commissions interpreted their tasks extensively by also advising the applicants on the development of the trial programs and sometimes extending them. The research projects of the SFB 112 were evaluated with respect to their ethical defensibility on the basis of 6 theses, which followed the principles of the World Medical Association and which were strictly observed in the leading clinical research institutes in the Federal Republic of Germany. They were in line with the standards required in the international framework, in particular by the World Medical Association and the National Institutes of Health.

## 5. Conclusion

As has been shown, the Ethics Commission of the University of Ulm was founded no earlier than July 14, 1971 and no later than the first half of February 1972. Most probably it was established due to extrinsic incentives, which were necessary for the funding of the SFB 112. The German Research Foundation played a decisive role in the establishment of the first ethics commissions in Germany. The universities had to create ethics commissions in order to be able to obtain additional funds from the Foundation for their research. Thus, the Foundation initiated the institutionalization of the ethics commissions in the early 1970s.

If we look at the first ethical commissions from an international perspective, we notice that the reasons for their establishment differed depending on the local context, but had similar features. The governmental funders of clinical research played an important role in stimulating the creation of the first ethics commissions. However, unlike in the US, in Germany the motivation of the German Research Foundation was driven by the experience of Nazi medical crimes and the fear of the decreasing sensitivity of researchers in ethics issues in the post-war years. Particularly after analyzing in detail the early years of the Ethics Commission of the University of Ulm, we conclude that its development took into account the international discourse on bioethics, but did not draw directly on the US experience. The composition of the first commissions was experimental and included representatives from various fields of medicine and science, as well as laypersons. Over time, the ethics commission included more representatives of medical branches. Thus, the creation of the earliest ethics commissions, their composition, and the direction of their work followed a practical path. The adoption of the Tokyo revision of the Helsinki Declaration in 1975 contributed to the widespread establishment of ethics commissions worldwide.

## Data availability statement

The data analyzed in this study is subject to the following licenses/restrictions: We used documents from the Archive of the Ethics Commission of the University of Ulm. Requests to access these datasets should be directed to FS, florian.steger@uni-ulm.de.

## Author contributions

FS and OK conceptualized and designed the study. FS collected the sources. All authors wrote the first draft of the manuscript, analyzed the sources as well as contributed to the manuscript revision, read, and approved the submitted version.

## Conflict of interest

The authors declare that the research was conducted in the absence of any commercial or financial relationships that could be construed as a potential conflict of interest.

## Publisher’s note

All claims expressed in this article are solely those of the authors and do not necessarily represent those of their affiliated organizations, or those of the publisher, the editors and the reviewers. Any product that may be evaluated in this article, or claim that may be made by its manufacturer, is not guaranteed or endorsed by the publisher.
